# Evaluation of Short-Chain Antimicrobial Peptides With Combined Antimicrobial and Anti-inflammatory Bioactivities for the Treatment of Zoonotic Skin Pathogens From Canines

**DOI:** 10.3389/fmicb.2021.684650

**Published:** 2021-08-11

**Authors:** Qiyu Tang, Chunyi Yang, Weitian Li, Yuhang Zhang, Xinying Wang, Weixin Wang, Zhiling Ma, Di Zhang, Yipeng Jin, Degui Lin

**Affiliations:** ^1^Department of Veterinary Clinical Science, College of Veterinary Medicine, China Agricultural University, Beijing, China; ^2^Laboratory of Anatomy of Domestic Animals, College of Veterinary Medicine, China Agricultural University, Beijing, China; ^3^Key Lab of Animal Epidemiology and Zoonosis of Ministry of Agriculture, College of Veterinary Medicine, China Agricultural University, Beijing, China; ^4^Modern Animal Research Center, Nanjing University, Nanjing, China; ^5^Research and Development Department, Artron BioResearch Inc., Vancouver, BC, Canada

**Keywords:** antimicrobial peptide, *Microsporum canis*, *Staphylococcus pseudintermedius*, anti-inflammation, anti-biofilm, mouse skin infection model, synergistic efficacy

## Abstract

The incidence of zoonotic *Staphylococcus pseudintermedius* and *Microsporum canis* infections is rapidly growing worldwide in the context of an increasing frequency of close contact between animals and humans, presenting challenges in both human and veterinary medicine. Moreover, the development of microbial resistance and emergence of recalcitrant biofilms, accompanied by the insufficiency of new antimicrobial agents, have become major obstacles in treating superficial skin infections caused by various microbes including *S. pseudintermedius* and *M. canis*. Over recent years, the prospects of antimicrobial peptides as emerging antimicrobials to combat microbial infections have been demonstrated. In our study, two novel short-chain peptides, namely, allomyrinasin and andricin B, produced by *Allomyrina dichotoma* and *Andrias davidianus*, were revealed to exhibit potent antimicrobial efficacy against clinical isolates of *S. pseudintermedius* and *M. canis* with remarkable and rapid fungicidal and bactericidal effects, while allomyrinasin exhibited inhibition of biofilm formation and eradication of mature biofilm. These peptides displayed synergistic activity when combined with amoxicillin and terbinafine against *S. pseudintermedius* and *M. canis*. Cytoplasmic leakage via cytomembrane permeabilization serves as a mechanism of action. Extremely low hemolytic activity and serum stability *in vitro*, as well as superior anti-infective efficacy in reducing bacterial counts and relieving the inflammatory response *in vivo*, were detected. The potent antibacterial, antifungal, and anti-inflammatory activities of allomyrinasin and andricin B might indicate promising anti-infective alternatives for the treatment of *S. pseudintermedius* and *M. canis* infections in the context of human and veterinary medicine.

## Introduction

In human history, the most challenging epidemics with pandemic potential, such as pestis, HIV, tuberculosis, SARS-2003, brucellosis, and COVID-19, are attributed to microbes that develop from their counterparts that naturally inhabit animals (Jones et al., 2008; Zhou et al., 2020). Animals have been reported as reservoirs for more than 60% of human infectious diseases, which is also the case for approximately 75% of emerging diseases. It is evident that the majority of human infectious diseases derive from animals (Taylor et al., 2001). Among them, zoonotic skin infection has attracted increasing interest in the fields of human and veterinary medicine, as well as animal husbandry.

Staphylococci have been associated with a plethora of human medical issues, including localized skin infections, surgical site infections (SSIs), and medical device-related infections (Yong et al., 2019). Notably, *Staphylococcus pseudintermedius* has appeared over the last decade as a critically significant bacterial pathogen responsible for skin, soft tissue, wound, and SSIs and possesses increasing zoonotic potential for cutaneous infections in humans (Murayama et al., 2013). *S. pseudintermedius* infections have been widely reported in recent years because of their potential for widespread transmission in human populations and significant implications for public health. This pathogen was blamed for apparent zoonotic infections in humans via close contact with affected animals, such as animals on farms or in household (Stegmann et al., 2010; Weese and van Duijkeren, 2010). Staphylococcal infections can result in local and systemic infections, extended inflammation, and delayed wound healing (Wolcott et al., 2010). Worryingly, recent studies have reported that the emergence of drug resistance of *S. pseudintermedius* against demonstrating the emergence of drug resistance to antimicrobial agents applied in Europe and North America, indicating the urgency of novel therapeutic alternatives (Perreten et al., 2010; Ong et al., 2014).

Apart from staphylococci, *Microsporum canis*, a worldwide distributed zoophilic dermatophyte, is another significant pathogen frequently related to integumentary and hair infections in humans and animals (Pasquetti et al., 2017). Animals can act as reservoirs and the major route for *M. canis* infection spreading in humans (Cafarchia et al., 2006). However, *M. canis* infections usually have a high recurrence rate and therefore require a long-cycle treatment. Medical therapy with conventional antifungal antimicrobials like triazole agents is prone to induce hepatotoxicity and nephrotoxicity (Kyriakidis et al., 2017). In light of the increasing frequency of *S. pseudintermedius* and complexity of treatment for *M. canis*, there is a compelling demand for new antimicrobial agents with novel mechanisms of action to reduce the incidence of bacterial resistance and drug toxicity.

Antimicrobial peptides (AMPs) have arisen as novel therapeutic alternatives against microbial infections in recent years. These peptides possess rapid and broad-spectrum antimicrobial activity against bacterial and fungal pathogens via membrane disruption or endocellular constituent binding, leading to a low potential for the development of resistance (Brunetti et al., 2017). In addition, they display an effective ability to prevent biofilm formation and to eradicate sessile communities of microbes. With the ability to modulate the host inflammatory response and prevent cellular toxicity, AMPs have shown significant potential for treating skin infections and accelerating the wound healing (Woodburn et al., 2019). However, several limitations of AMPs, including proteinase degradation, serum binding, and high cost of production (Sharma et al., 2018), have substantially stunted their pharmaceutical utilization. Therefore, exploration of short-chain AMPs with antibacterial and antifungal potency, synergism with conventional therapeutics, and suitability for animal application will be a direction for future development.

Several studies have reported the efficacy of certain types of AMPs in killing *S. pseudintermedius* and *M. canis in vitro* (Santoro and Maddox, 2014; Simonetti et al., 2014); however, there are little data about the application of newly discovered AMPs to *in vitro* antimicrobial assays for clinical isolates of *S. pseudintermedius* and *M. canis* from China and their activity in a *S. pseudintermedius*-induced mouse skin infection model. Two short synthetic peptides, namely, allomyrinasin and andricin B, identified from *Allomyrina dichotoma* and *Andrias davidianus*, and three other original short-chain peptides have been previously described as effective against staphylococcal and fungal strains, which may serve as good candidates for further research (Pei et al., 2018; Bao et al., 2019; Bessa et al., 2019; Chaparro-Aguirre et al., 2019; Lee et al., 2019). In our present study, we confirmed that allomyrinasin and andricin B exhibited potent antimicrobial activities by the disruption of microbial cell membrane. In addition, we evaluated the antibiofilm activities of the peptides and investigated synergistic activities with conventional antimicrobials. The toxicity of these peptides to mammalian erythrocytes and their antimicrobial stability in serum were assessed to identify their potential for animal application. Moreover, the mouse model of *S. pseudintermedius* skin infection was established; and bacterial counts, histopathological techniques, and Western blotting were performed to explore the antibacterial and anti-inflammatory efficacy of allomyrinasin and andricin B *in vivo*.

## Materials and Methods

### Peptides

**FIGURE 1:**
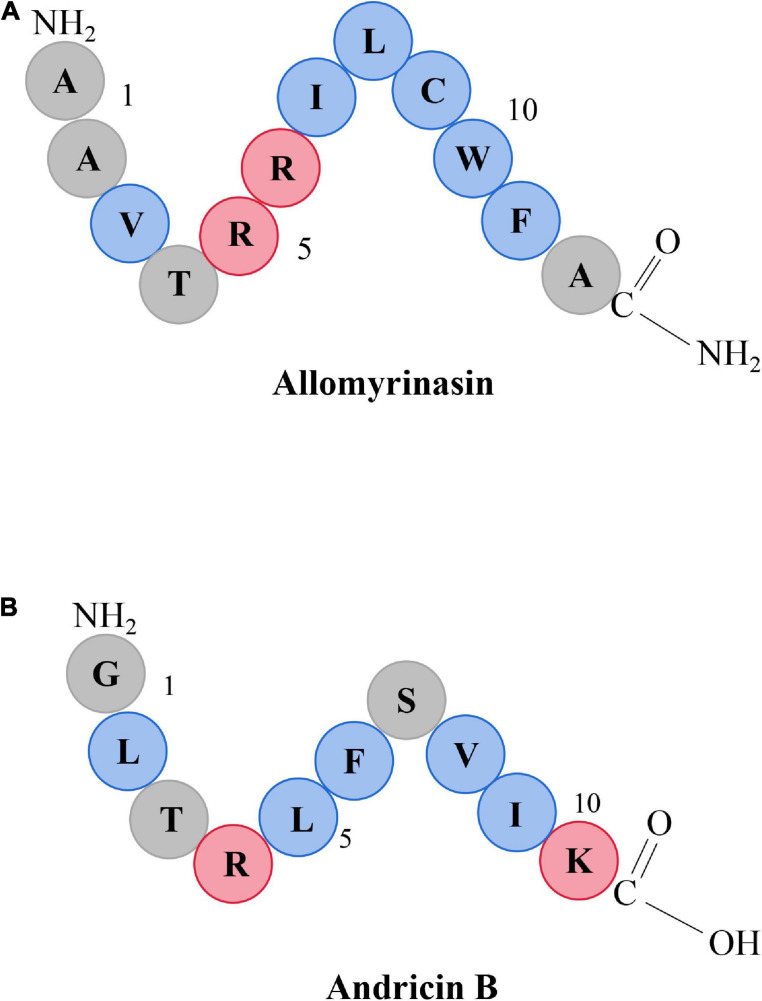
Schematic representation of allomyrinasin **(A)** and andricin B **(B)**. Hydrophobic residues are in blue, while those residues with a net positive charge are in red.

The characteristics of five AMPs, allomyrinasin (AAVTRR ILCWFA-NH_2_) (Figure 1A), andricin B (GLTRLFSVIK) (Figure 1B), pinipesin (VAEARQGSFSY), nigrocin-HLM (GLLS GILGAGKKIVF), Hs02 (KWAVRIIRKFIKGFIS), and nisin (ITSISLCTPGCKTGALMGCNMKTATCHCSIHVSK), were described previously (Rogers, 1928; Pei et al., 2018; Bao et al., 2019; Bessa et al., 2019; Chaparro-Aguirre et al., 2019; Lee et al., 2019). Allomyrinasin and andricin B are cationic AMPs identified from *A. dichotoma* and *A. davidianus* blood, respectively. These short-chain peptides possess net positive charge of +3 and +2, as depicted in the schematic in Figure 1. Pinipesin, nigrocin-HLM, and Hs02 are another three novel AMPs and exhibit potent antimicrobial effects against a wide spectrum of microorganisms, simultaneously with low hemolytic and cytotoxic activities. All peptides in this study were obtained by chemical synthesis using a standard solid-phase 9-fluorenylmethoxycarbonyl (Fmoc) and refined by reverse-phase high-performance liquid chromatography (RP-HPLC) (GL Biochem, Shanghai, China). A purity greater than 99.6% was identified by liquid chromatography–mass spectrometry (LC-MS) (Oddo et al., 2015).

### Pathogens

Bacterial strains included in the study were isolated from clinical specimens obtained from the skin of dogs with pyoderma at the Small Animal Protection Center (Hainan, China), and fungal strains were acquired from the skin of dogs admitted to the Veterinary Teaching Hospital of China Agricultural University (Table 1). Bacterial and fungal strains were examined at the Diagnostic Laboratory of China Agricultural University by 16S rRNA gene amplification and rDNA ITS sequence analysis by polymerase chain reaction (PCR), respectively. The amplified PCR products were then electrophoresed and sequenced by the Sanger method at Majorbio Sanger Bio-pharm Technology (Beijing, China). The data of sequencing analysis were submitted to the GenBank database of National Center for Biotechnology Information (NCBI, Bethesda, MD, United States).

**TABLE 1:** Minimum inhibitory concentrations and minimum bactericidal/fungicidal concentrations (MICs and MBCs/MFCs, μg/ml) of selected peptides against clinical isolates of planktonic bacteria and dermatophytes.

Peptides Bacterial strains	Allomyrinasin		Andricin B		Pinipesin		Nigrocin-HLM		Hs02	
	MIC	MBC/MFC	MIC	MBC/MFC	MIC	MBC/MFC	MIC	MBC/MFC	MIC	MBC/MFC
**Gram-positive isolates**										
*Staphylococcus pseudintermedius* MW767056	8	8	32	32	128	128	>256	>256	>256	>256
*S. pseudintermedius* MW793383	16	16	32	32	64	64	256	256	256	256
*S. pseudintermedius* MW793384	8	8	128	128	128	128	128	128	128	128
*S. pseudintermedius* MW793385	8	8	64	64	256	256	128	128	256	256
*S. pseudintermedius* MW793386	16	16	64	64	256	256	>256	>256	>256	>256
*S. pseudintermedius* MW793387	32	32	128	128	>256	>256	>256	>256	>256	>256
*S. pseudintermedius* MW793388	8	8	64	64	128	128	32	32	16	16
*S. pseudintermedius* MW793389	8	8	128	128	256	256	128	128	32	32
*S. pseudintermedius* MW793390	16	16	128	128	64	64	128	128	128	128
*S. pseudintermedius* MW793391	8	8	32	32	128	128	256	256	256	256
*S. pseudintermedius* MW793392	8	8	64	64	64	64	256	256	128	128
*S. pseudintermedius* MW767046	8	8	32	32	128	128	128	128	64	64
*Staphylococcus cohnii* MW767053	32	32	64	64	>256	>256	128	128	32	32
*Staphylococcus haemolyticus* MW767051	>256	>256	>256	>256	>256	>256	64	64	128	128
*Staphylococcus sciuri* MW767055	>256	>256	>256	>256	>256	>256	>256	>256	>256	>256
*Staphylococcus simulans* MW767052	128	128	256	256	128	128	16	16	64	64
**Gram-negative isolates**										
*Proteus mirabilis* MW767054	>256	>256	>256	>256	>256	>256	64	64	128	128
**Dermatophyte**										
*Microsporum canis* MW767025	0.5	0.5	1	1	1	1	4	4	2	2
*M. canis* MW767026	0.5	0.5	0.5	0.5	1	1	2	2	4	4
*M. canis* MW767027	1	1	1	1	1	1	2	2	2	2
*M. canis* MW768151	0.5	0.5	1	1	0.5	0.5	1	1	1	1
*Trichophyton mentagrophytes* MW766984	2	2	1	1	0.5	0.5	2	2	1	1
*Microsporum gypseum* MW766983	0.5	0.5	2	2	2	2	1	1	0.5	0.5

### Antimicrobial Assays

The broth microdilution technique was applied to measure the minimal inhibitory concentrations (MICs) of the tested peptides (Wiegand et al., 2008). In brief, the concentrations of bacterial strains (MW767051–MW793392) were diluted to 1 × 10^5^ CFU/ml in Mueller–Hinton (MH) broth (MHB) and added to a twofold serial dilution of peptides ranging from 1 to 512 μg/ml in 96-well microplates. The MIC values were recorded as the lowest concentration preventing visible bacterial growth after 24 h of incubation at 37°C. Next, 10 μl of overnight suspension cultivated on a MH plate was performed with colony counting for determination of minimum bactericidal concentrations (MBCs). The lowest concentrations with no evidence of colonies were considered MBCs. Similarly, a twofold serial dilution of peptides varying from 0.25 to 16 μg/ml was added to the fungal suspension (MW766983–MW767027) at concentrations of 1 × 10^3^ CFU/ml in RPMI-1640 (Gibco, Grand Island, NY, United States). The culture was observed daily, and the MICs were determined at 7 days after incubation as the lowest concentration with no microbial growth at 30°C. Next, 100 μl of the fungal culture was smeared on Sabouraud dextrose agar (SDA) plates to determine the minimal fungicidal concentration (MFC), considered the lowest concentration of drug inhibiting 100% growth. Experiments were run in triplicate.

### Time-Kill Assays

The microbial killing activity of allomyrinasin and andricin B against *S. pseudintermedius*, *Staphylococcus cohnii* and *M. canis* strains (MW767056, MW767053, and MW768151) was determined based on prior antimicrobial susceptibility assays. Briefly, bacteria and dermatophytes were incubated aerobically overnight at 37°C in MHB and RPMI-1640 media and then diluted to 1 × 10^5^ CFU/ml. Peptides (at 0.5×, 1×, 2×, and 4× MIC) and equivalent saline were applied to inocula with rotation speed at 250 r.p.m. After 0, 15, 30, 60, 120, 180, and 300 min, 100 μl of aliquots was removed for 10-fold serial dilution and then plated on brain heart infusion (BHI) and SDA plates for viable counts in triplicate (Blondeau et al., 2012; Simonetti et al., 2014).

### Anti-biofilm Assays

Minimal biofilm inhibitory concentration (MBIC) and minimal biofilm eradication concentration (MBEC) assays were performed in accordance with assays utilizing prototype tetrazolium salt, 2,3,5-triphenyl-tetrazolium chloride (TTC) or 2,3-bis (2-methoxy-4-nitro-5-sulfophenyl)-5-[(phenylamino)carbonyl]-2*H*-tetrazolium hydroxide (XTT) (Delattin et al., 2014; Sabaeifard et al., 2014). *S. pseudintermedius* MW767056, *S. cohnii* MW767053, and *M. canis* MW768151 strains were applied in these assays. Briefly, a suspension of overnight inoculum, rinsed with phosphate-buffered saline (PBS), was adjusted to 1 × 10^6^ CFU/ml with culture media and then incubated with peptides of the same concentration range applied in the MIC assay in a round-bottomed 96-well microplate for MBIC determination. Finally, biofilms were washed and quantified after 24 h of static incubation at 37°C.

Microbial biofilm eradication was determined using a previously described protocol. One hundred microliters of aliquots of overnight culture diluted to 10^6^ CFU/ml was added to a round-bottomed 96-well microplate and then incubated at 37°C for 3 h for adhesion, followed by the removal of planktonic cells with PBS and the addition of 100 μl of fresh media. After 48 h of static incubation at 37°C for biofilm maturation, mature biofilms were washed and incubated with a twofold serial dilution series of peptides at 37°C for 24 h. Following sufficient growth, inocula were rinsed with PBS and quantified with TTC and XTT. Evaluations were done in triplicate.

### Combination Therapy Analysis

Allomyrinasin and andricin B were investigated for their interaction with antibiotics and antifungals such as amoxicillin (a major drug for *Staphylococcus*-induced skin infection) (Summers et al., 2014) and terbinafine hydrochloride (a main drug for *M. canis*-induced dermatophytosis) (Aneke et al., 2021). *S. pseudintermedius* MW767056, *S. cohnii* MW767053, and *M. canis* MW768151 were applied as tested strains in this assay. Overnight cultures were diluted to a final concentration of 1 × 10^5^ CFU/ml in a total of 200 μl. The effect of combined medication was determined by testing a twofold serial dilution series of antimicrobials in combination with a constant concentration (1/4 × MIC) of peptides, which was unable to inhibit microbial growth independently. This assay was carried out in triplicate. A fractional inhibitory concentration (FIC) index was calculated to indicate the underlying interaction between two antimicrobials, and interpretation of the interaction type was performed as follows.

A FIC was derived for each well containing the lowest inhibitory combination of antimicrobials from the following calculation:

$$\begin{array}{c} \mathrm{FIC}\mathrm{of}\\ \mathrm{antimicrobial}A \end{array}=\frac{\mathrm{MIC}\mathrm{of}\mathrm{antimicrobial}A\mathrm{in}\mathrm{combination}}{\mathrm{MIC}\mathrm{of}\mathrm{antimicrobial}A\mathrm{alone}},$$

$$\begin{array}{c} \mathrm{FIC}\mathrm{of}\\ \mathrm{antimicrobial}B \end{array}=\frac{\mathrm{MIC}\mathrm{of}\mathrm{antimicrobial}B\mathrm{in}\mathrm{combination}}{\mathrm{MIC}\mathrm{of}\mathrm{antimicrobial}B\mathrm{alone}}.$$

A _FIC  index=FIC  of  antimicrobial  A  +  FIC  of  antimicrobial  B_. Synergism and antagonism presented as FIC indices less than 0.5 and more than 4, respectively. All values ranging from 0.5 to 4.0 suggested an indifferent interaction, except for an additive effect, classified as a FIC index equivalent to 1 (Yan and Hancock, 2001).

### Bacteriolysis Analysis

Cytolysis, indicated by a reduction in the absorbance at 600 nm, was identified as described previously (Oliva et al., 2003). Briefly, overnight culture of *S. pseudintermedius* MW767056 incubated in MHB at 37°C with an OD600 of ≈0.6 was diluted to 1 × 10^7^ CFU/ml. Aliquots (100 ml) were placed in 96-well microplates, followed by the addition of allomyrinasin and andricin B at 4× MIC. Nisin at 4× MIC and 0.9% NaCl (w/v), normal saline, were used as the positive and negative controls, respectively. Turbidity was recorded every 2 h until 10 h by a microplate reader at 600-nm absorbance. The assay was carried out in triplicate.

### Calcein Leakage Analysis

The cell membrane permeabilization induced by peptides was measured by variations in preloaded calcein leakage from lysed cells as described previously (Xiong et al., 2005). In brief, the inocula of *S. pseudintermedius* MW767056 were cultivated in MHB at 37°C overnight, collected by centrifugation, rinsed with PBS, and then diluted to 10^8^ CFU/ml. After incubation with 5 μM of calcein for 2 h at 37°C, cells, gathered by rinses and centrifugation, were diluted to a suspension of 10^5^ CFU/ml. Then, 100 μl of aliquots was placed in black 96-well microplates, followed by the addition of allomyrinasin and andricin B at a series of concentrations of 0.5, 1, 5, and 10× MIC. Bacterial cultures treated with 0.9% NaCl (w/v) and nisin at 10× MIC were defined as the negative and positive controls, respectively. Leakage was monitored every 10 min for up to 1 h using a fluorescence plate reader. Membrane permeabilization (%) was determined as the absolute percent calcein leakage in peptide-treated samples in contrast to peptide-untreated samples. Experiments were done in triplicate and repeated independently twice.

### Hemolytic Activity

The hemolysis assay was carried out by inoculating mammalian erythrocytes obtained from fresh defibrinated sheep and mouse blood (Thermo Fisher Scientific, Waltham, MA, United States) with the peptides. Briefly, peptides were incubated at 37°C with a 4% erythrocyte suspension with a twofold serial dilution series of peptides ranging from 512 to 1 μg/ml for 2 h. PBS and 1% nonionic detergent Triton X-100^TM^ (Sigma-Aldrich, St. Louis, MO, United States) diluted in PBS served as negative and positive controls, respectively. Cytolysis was evaluated by the measurement of absorption using a microplate reader at 550 nm. Experiments were done in triplicate. The hemolysis is derived from the following calculation:

$$\mathrm{Haemolysis}\%=\frac{\text{A-APBS}}{\text{ATriton}-A\text{PBS}}\times 100\%.$$

A represents the absorbance of samples treated with peptide, while A_PBS_ and A_Triton_ indicate the absorbance of the negative and positive controls, respectively.

### Antimicrobial Activities in Serum

Peptide stability was identified as the variation in antimicrobial activity after inoculation with serum. In this assay, *S. pseudintermedius* MW767056, *S. cohnii* MW767053, and *M. canis* MW768151 were applied as tested strains. Twenty-five percent fetal bovine serum (FBS) diluted in RPMI-1640 medium was stored at 37°C for 15 min for pre-equilibration and then inoculated with peptides with a twofold serial dilution of concentrations from 512 to 1 μg/ml. After incubation at 37°C for 4 h, peptides were collected by centrifugation, and 50 μl of aliquots was added to the same amount of culture at a concentration of 1 × 10^5^ CFU/ml in 96-well microplates. The MICs were defined as the lowest concentration of peptides preventing any discernible growth after incubation with serum. All the peptide concentrations and controls had three replicates in a single 96-well plate, and two independent experiments were performed.

### *In vivo* Mouse Skin Infection Model

All experiments on animals were employed in accordance with the Chinese Regulations for Laboratory Animals—The Guidelines for the Care of Laboratory Animals and Laboratory Animal Requirements of Environment and Housing Facilities (GB 14925-2010, National Laboratory Animal Standardization Technical Committee). The animal studies and research protocols were authorized by the Animal Care and Use Committee of China Agricultural University. The murine skin abrasion model was established using overnight culture of *S. pseudintermedius* (Abe et al., 1992). Forty specific pathogen-free (SPF), 6- to 8-week-old female BALB/c mice weighing 15–21 g were obtained from Charles River Laboratories (Beijing, China). All mice were raised singly in cages under a 12-h light/dark cycle and allowed *ad libitum* access to feed and water. Skin abrasion wounds of 3 cm^2^ were performed using sand paper on the shaved dorsal skin after anesthetization with 2.5% isoflurane. Damage was confined to the epidermis, and bleeding was carefully avoided. Fifty microliters of *S. pseudintermedius* MW767056 suspension at concentrations of 10^8^ CFU/ml was spread on scratched skin areas loaded with a total inoculum of 5 × 10^6^ CFU. Four hours after inoculation, 40 mice were randomly divided into four groups (three antimicrobial treatment groups and one infected control group treated with 50 μl 0.9% NaCl w/v). Treatment groups were topically treated daily for 3 days with 50 μl of allomyrinasin, andricin B, or amoxicillin at 6× MIC with direct application onto the infected skin, followed by the placement of a transparent film dressing of 3 cm^2^ (Tegaderm^TM^, 3M, St. Paul, MN, United States). The dosage of AMPs was determined by *in vitro* hemolysis study, which indicated the minimum hemolytic concentration (MHC), required to cause 10% hemolysis, was between 128 and 256 μg/ml and around 512 μg/ml for allomyrinasin and andricin B, respectively. In order to minimize hemolytic effect induced by peptides on mice and to simultaneously not negatively influence the therapeutic effects, 6× MIC of allomyrinasin and andricin B was chosen as the dosage for application. Body weight, clinical observations, and skin wound areas were also recorded daily. On the first and third days of administration, five mice were randomly removed from each group for euthanasia after anesthetization with 2.5% isoflurane. The infected skin and liver tissues were harvested separately for further analysis with a portion homogenized for bacterial culture and Western blotting analysis and another portion fixed in 4% formalin for histopathological assessment. Treatment efficacy was determined according to wound healing, inflammatory response, and bacterial load in skin. Diluted samples for bacterial culture were added and spread in BHI plates incubated at 37°C for 24 h following viable bacterial counting. Evaluation of interleukin-6 (IL-6) and tumor necrosis factor-α (TNF-α) protein expression in infected skin was performed by Western blotting as described previously (Tang et al., 2017).

### Histopathological Examination

The treatment segments of dorsal skin tissues were cut out, rinsed with sterile physiologic saline, and then fixed in 4% paraformaldehyde. The formalin-fixed tissues, embedded in paraffin, were sectioned at 5-μm thickness and then stained with hematoxylin and eosin (Qi et al., 2016). Three sections of each tissue were evaluated.

### Western Blotting Analysis

To examine the expression of IL-6 and TNF-α at the protein level, immunoblotting was performed as reported previously (Tang et al., 2017). Proteins from dorsal skin tissues were extracted for Western blotting assays. Primary antibodies included anti-IL-6 (1:1,000, Abcam, Waltham, MA, United States) and anti-TNF-α (1:1,000, Abcam, United States). To verify equal sample loading, the membrane was incubated with rabbit polyclonal anti-β-actin antibody (1:1,000, Abcam, United States) as an internal control. Horseradish peroxidase-conjugated goat anti-rabbit IgG (1:5000, Abcam, United States) was used as the secondary antibody. The membranes were exposed under a chemiluminescent imaging analysis system (GE Healthcare Life Science, Danderyd, Sweden). Samples not treated with primary antibodies were considered negative controls. Relative levels of each protein were normalized to those of β-actin in each sample.

### Statistical Analysis

Statistical analysis of data was performed with SPSS 22 (Systat Software, Erkrath, Germany) by one-way ANOVA followed by Tukey’s multiple comparisons test and presented as the means ± SD. Log transformations were performed as needed to maintain homogeneity of variance and normality. *p*-Values lower than 0.05 were considered indicative of statistical significance.

## Results

### Strain Identification

The identification of bacterial and fungal isolates using Sanger method confirmed that the strains studied in this work were *S. pseudintermedius*, *S. cohnii*, *Staphylococcus haemolyticus*, *Staphylococcus simulans*, *Proteus mirabilis* and *Staphylococcus sciuri*, *M. canis*, *Microsporum gypseum*, and *Trichophyton mentagrophytes*. Among all isolates, 12 were identified as *S. pseudintermedius* and four were *M. canis*. Upon submission of the bacterial 16S rRNA and fungal ITS sequences to the NCBI GenBank database, the following accession numbers have been assigned: MW766983–MW793392 (Supplementary Table 1).

### Antimicrobial Assays

The peptides exerted microbicidal effects on the tested bacteria and fungi but to different degrees (Table 1). Allomyrinasin and andricin B possessed comparatively strong bioactivity against *S. pseudintermedius* (MIC and MBC of 8 and 32 μg/ml, respectively). In contrast, pinipesin, nigrocin-HLM, and Hs02 showed relatively weak bioactivity against *S. pseudintermedius*, and their MIC and MBC values were 128 μg/ml, > 256μg/ml, and > 256μg/ml, respectively. In addition to pinipesin and nigrocin-HLM, allomyrinasin, andricin B, and HS02 were also potent against *S. cohnii* (MIC and MBC of 32, 64, and 32 μg/ml, respectively). Allomyrinasin, andricin B, and pinipesin were moderately inhibitory against *S. simulans*, but no obvious inhibitory effect was observed against *S. haemolyticus* and *P. mirabilis*. Nigrocin-HLM and Hs02 exhibited strong potency against *S. simulans* and moderate inhibitory activity against *S. haemolyticus* and *P. mirabilis* with MICs and MBCs of 128 μg/ml. No antibacterial activity of peptides against *S. sciuri* was detected. Interestingly, all of the tested peptides had strong antifungal activities against *M. canis*, *M. gypseum*, and *T. mentagrophytes*, with MIC values varying from 0.5 to 2 μg/ml. Considering the potent antimicrobial effect of allomyrinasin and andricin B on *S. pseudintermedius*, *S. cohnii*, and *M. canis*, we selected them as the tested targets to perform the following antimicrobial assays.

### Microbial Killing Kinetics

**FIGURE 2:**
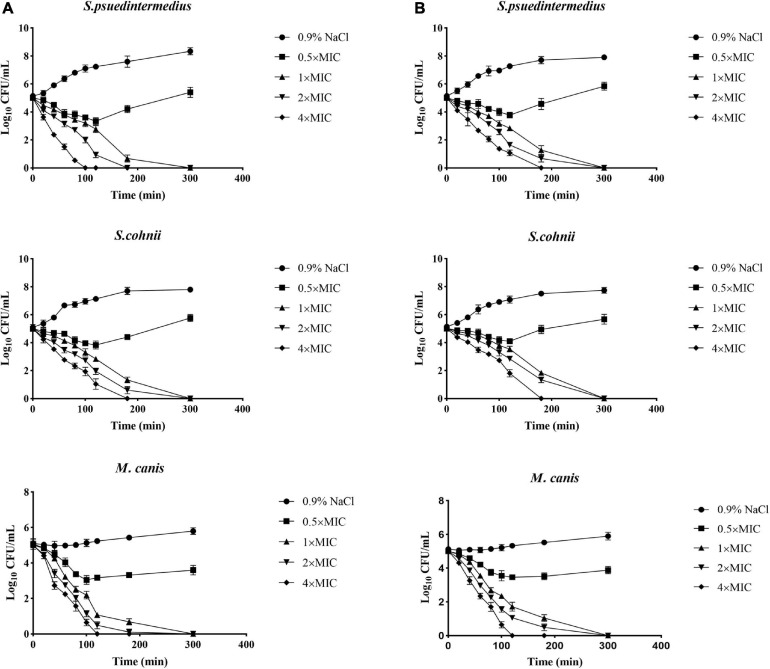
Microbial killing kinetics of allomyrinasin **(A)** and andricin B **(B)** against *Staphylococcus pseudintermedius* MW767056, *Staphylococcus cohnii* MW767053, and *Microsporum canis* MW768151 at four different concentrations. Inocula treated with physiological saline (0.9% NaCl) were used as a control. The error bars represent the standard deviation (SD) for three repeats.

The excellent antimicrobial activity of allomyrinasin and andricin B against clinical isolates of *S. pseudintermedius*, *S. cohnii*, and *M. canis* was identified; and next, the killing kinetics of these two AMPs were examined. Both peptides exhibited complete eradication of these strains in a time- and concentration-dependent manner (Figure 2). Allomyrinasin displayed rapid bactericidal efficacy and has the capability of totally clearing an inoculum of *S. pseudintermedius* (1 × 10^5^ CFU/ml) within 100 and 180 min at 4× and 2× MIC, respectively, but more moderate bactericidal effects were observed, presented as the requirement of more time for complete elimination of *S. cohnii*. At a low concentration of 0.5× MIC, allomyrinasin could only inhibit the growth of *S. pseudintermedius* and *S. cohnii*, and the bacteria began to grow in large quantities after 3 h.

In contrast to allomyrinasin, andricin B demonstrated relatively strong killing kinetics with the complete elimination of a starting inoculum of *S. cohnii* (1 × 10^5^ CFU/ml) within a range from 180 to 300 min at 4×, 2×, and 1× MIC. For the *S. cohnii* strain, andricin B showed slower bactericidal activity against *S. pseudintermedius*. At a low concentration of 0.5× MIC, obvious inhibitory efficacy against *S. pseudintermedius* and *S. cohnii* was not detected.

Interestingly, allomyrinasin and andricin B displayed potent fungicidal activity at >0.5× MIC within 2 h. These peptides completely killed the initial inoculum at different concentrations within 5 h. The results show that allomyrinasin and andricin B exerted rapid and strong fungicidal effects on *M. canis*.

### Biofilm Disruption

**FIGURE 3:**
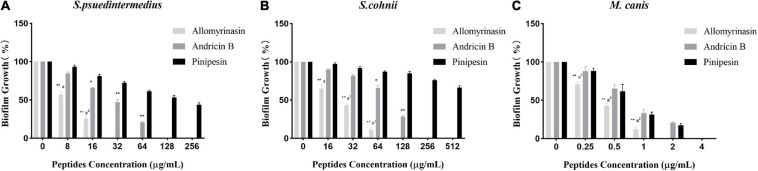
Effect of antimicrobial peptides on biofilm formation rate of *Staphylococcus pseudintermedius* MW767056 **(A)**, *Staphylococcus cohnii* MW767053 **(B)**, and *Microsporum canis* MW768151 **(C)**. All data are shown as the mean ± SD from the experiment performed in triplicate. *p < 0.05, **p < 0.01 significant difference between pinipesin and the other two peptides; ^a^*p* < 0.05, ^a2^*p* < 0.01 significant difference between allomyrinasin and andricin B.

Microbial biofilms have posed serious challenges on account of their role in recurrent infections and drug resistance. In our study, the tested peptides were able to eradicate mature biofilms and prevent their formation (Table 2). Allomyrinasin maintained potent elimination of established biofilms and inhibition of the sessile cells of *S. pseudintermedius* and *M. canis* (MBIC of 32 and 2 μg/ml, respectively) with remarkable increases in MBEC values (128 and 8 μg/ml, respectively) but showed a moderate inhibitory effect on biofilm formation by *S. cohnii*. Compared with andricin B and pinipesin, allomyrinasin at concentrations higher than or equal to 8 and 0.25 μg/ml began to exhibit an obvious inhibitory effect on bacterial biofilm growth by *S. pseudintermedius* and *M. canis* (Figures 3A,C). In contrast, andricin B showed a weak effect on the formation of biofilms by *S. pseudintermedius* and *S. cohnii* with relatively high MBIC and MBEC values (MBIC of 128 and 256 μg/ml; MBEC of 256 and >256 μg/ml, respectively), but similar to allomyrinasin, andricin B completely inhibited biofilm formation and eradicated mature biofilms of *M. canis* exhibiting MBIC and MBEC of 4 and 16 μg/ml, respectively. A concentration ≥16 μg/ml had a more obvious inhibitory effect than allomyrinasin and pinipesin on bacterial biofilm growth by *S. cohnii* (Figure 3B). No obvious inhibition of pinipesin on the biofilm of *S. pseudintermedius* and *S. cohnii* was found; however, pinipesin exerted a strong effect on the biofilm formation and mature biofilm of *M. canis* with MBIC and MBEC of 4 and 16 μg/ml, respectively.

**TABLE 2:** The effects of allomyrinasin, andricin B, and pinipesin on mature biofilms and their formation by *Staphylococcus pseudintermedius* MW767056, *Staphylococcus cohnii* MW767053, and *Microsporum canis* MW768151.

Peptides	*S. pseudintermedius* MW767056		*S. cohnii* MW767053		*M. canis* MW768151	
	MBIC	MBEC	MBIC	MBEC	MBIC	MBEC
						μg/ml
Allomyrinasin	32	128	128	256	2	8
Andricin B	128	256	256	>256	4	16
Pinipesin	>256	>256	>256	>256	4	16

*MBIC, minimal biofilm inhibitory concentration; MBEC, minimal biofilm eradication concentration.*

### Synergistic Activity Analysis

The *in vitro* antimicrobial assays indicated that the tested peptides had the potential to be applied independently. Thus, there is a necessity to assess synergism between peptides and conventional therapeutics widely used in skin infections to optimize dosage regimens. The synergistic interaction of allomyrinasin and andricin B with conventional antibiotics and antifungals against *S. pseudintermedius*, *S. cohnii*, and *M. canis* isolates was evaluated. Allomyrinasin and andricin B exhibited synergistic activity in combination against *S. pseudintermedius*, *S. cohnii*, and *M. canis*, with FIC indices ranging from 0.3125 to 1.25. Andricin B exhibited synergistic activity with amoxicillin and terbinafine hydrochloride against *S. cohnii* and *M. canis*, with FIC indices ranging from 0.375 to 1.25. Interestingly, allomyrinasin was shown to be superior to andricin B, as potent synergism with amoxicillin and terbinafine hydrochloride was observed, with FIC indices varying from 0.3125 to 0.50. Most notably, only one indifferent interaction between andricin B and amoxicillin against *S. pseudintermedius* was detected (Table 3).

**TABLE 3:** The FIC index of the tested peptides in combination with amoxicillin and terbinafine.

	FIC index					
	*Staphylococcus pseudintermedius* MW767056		*Staphylococcus cohnii* MW767053		*Microsporum canis* MW768151	
Antimicrobials	Allomyrinasin	Andricin B	Allomyrinasin	Andricin B	Allomyrinasin	Andricin B
Allomyrinasin		0.3125		0.50		0.375
Andricin B	0.3125		0.50		0.375	
Amoxicillin	0.281	1.25	0.3125	0.50		
Terbinafine					0.375	0.50

*FIC, fractional inhibitory concentration.*

### Membrane Disruption Activity

**FIGURE 4:**
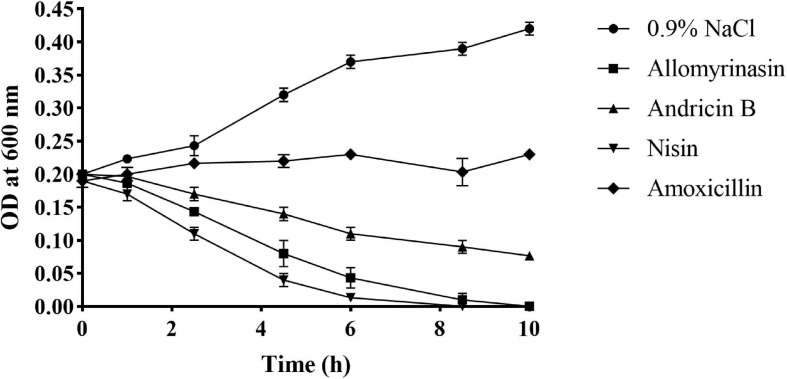
Bacterial killing kinetics of *Staphylococcus pseudintermedius* MW767056 culture treated with 4× minimal inhibitory concentration (MIC) allomyrinasin, andricin B, and amoxicillin by measuring absorbance at 600 nm over time. Nisin and saline served as positive and negative controls, respectively. All data represent the mean ± SD from the experiments performed in triplicate.

Rapid cytolysis is an indication of damage to the membrane by AMPs (Boman et al., 1993; Park et al., 1998). The membrane-destroying activities of peptides were evaluated by culture turbidity measurement. The turbidity of inocula treated with 4× MIC peptides was monitored by a microplate reader at OD600 over the course of 10 h. The results revealed that both allomyrinasin and andricin B led to a rapid reduction in the total bacterial count in inocula with a high concentration of 10^8^ CFU/ml. Allomyrinasin resulted in >30% and 75% decreases in absorbance after 2.5 and 6 h, respectively, while the data produced by andricin B were similar to those by allomyrinasin but to a lower degree. Nisin (4× MIC), a well-known membrane-perturbing peptide (Ruhr and Sahl, 1985), induced cell lysis at a higher rate than allomyrinasin, resulting in a >60% reduction in turbidity after 2.5 h. In contrast, the variations in turbidity of samples treated with 4× MIC amoxicillin, a cell wall synthesis-inhibiting antibiotic, were not recorded (Figure 4).

### Membrane Permeabilization

**FIGURE 5:**
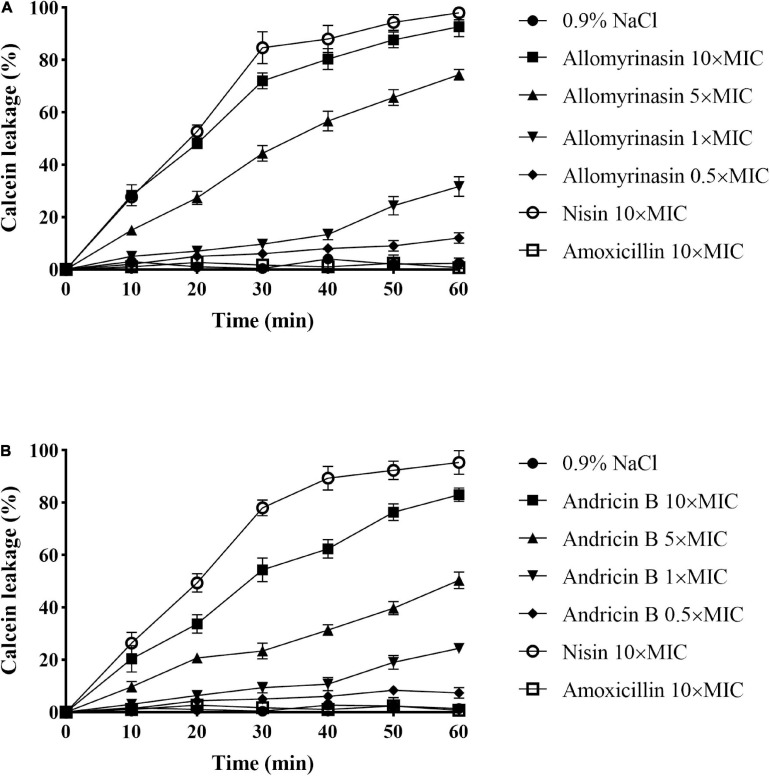
Cytoplasmic membrane permeabilization of *Staphylococcus pseudintermedius* MW767056 as a consequence of actions of allomyrinasin **(A)** and andricin B **(B)** at 0.5, 1, 5, and 10× minimal inhibitory concentration (MIC) μg/ml, suggested by calcein leakage over 1 h of exposure. All data are shown as the mean ± SD from two independent experiments performed in triplicate.

The calcein leakage assay was applied to assess the membrane permeabilization of the tested peptides as described previously (Xiong et al., 2005). Calcein leakage from cells indicated membrane integrity damage, represented as a decrease in fluorescence intensity. The tested peptides damaged the bacterial cell membrane, resulting in intracellular calcein leakage in a time- and concentration-dependent manner. The greater efficiency and potency of allomyrinasin than andricin B in membrane perturbation were recorded. At a concentration of 0.5× MIC, peptides resulted in calcein leakage lower than 10%, while peptides with a concentration of 1× MIC caused more than 20% leakage. Peptides brought about at least 70% and 50% leakage, respectively, within 1 h at a concentration of 5× MIC. When concentrations increased to 10× MIC, an obvious membrane damage reaction was detected for both peptides. More than 95% and 80% decreases in fluorescence intensity were measured for the two tested peptides. Nisin (10× MIC) led to at least 95% calcein leakage from cells, while amoxicillin exerted no obvious disruptive reaction on membrane integrity at a high concentration of 10× MIC, as expected (Figure 5).

### Hemolytic Activity

**FIGURE 6:**
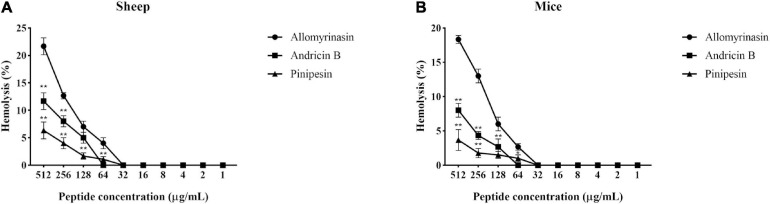
The hemolytic reaction was observed in 4% suspensions of sheep **(A)** and mouse **(B)** erythrocytes treated with an increasing peptide concentration. All data recorded after incubation for 2 h are shown as the mean ± SD from the experiment performed in triplicate. Triton X-100 (2%) and phosphate-buffered saline (PBS) served as positive and negative controls, respectively. Statistically significance was performed in comparison with allomyrinasin (*p < 0.05; **p < 0.01), using one-way ANOVA followed by Tukey’s analysis.

With the increase in the concentration of the tested peptides, the relative hemolysis rate also rose (Figure 6). Allomyrinasin, andricin B, and pinipesin showed slight hemolytic activity at high concentrations (>64 μg/ml) but displayed little or no obvious hemolytic activity at lower concentrations ranging from 1 to 64 μg/ml, which is closely associated with their MICs against microbes. Compared with those of allomyrinasin and andricin B, the relative hemolysis rates of pinipesin were still lower at high concentrations (>64 μg/ml), and no obvious hemolysis was observed at higher concentrations, which may be ascribed to their moderate antimicrobial bioactivities.

### Antimicrobial Activities in Serum

**FIGURE 7:**
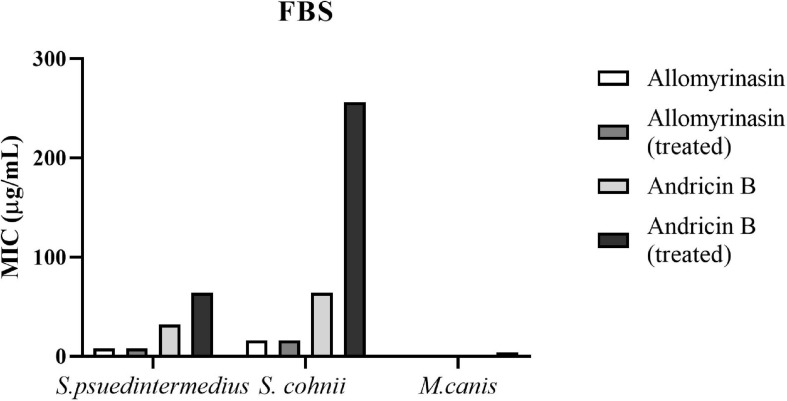
Minimal inhibitory concentrations (MICs) of allomyrinasin and andricin B with or without serum treatment against *Staphylococcus pseudintermedius* MW767056, *Staphylococcus cohnii* MW767053, and *Microsporum canis* MW768151. The experiments are carried out in triplicate from two independent experiments.

Both allomyrinasin and andricin B exhibited stable antimicrobial effects on *S. pseudintermedius*, *S. cohnii*, and *M. canis* in the presence of serum (Figure 7), with no obvious functional changes noticed. After treatment with FBS for 4 h, allomyrinasin maintained stable antimicrobial activity against *S. pseudintermedius*, *S. cohnii*, and *M. canis*. Similar to the MIC, MBIC, and MBEC results, the MIC of serum-incubated andricin B was also higher than that of allomyrinasin, but its bioactivity decreased to a certain extent. The MIC values of andricin B serum against *S. pseudintermedius*, *S. cohnii*, and *M. canis* increased from 32 to 64, 64 to 256, and 0.5 to 8 μg/ml, respectively. The biological activity of andricin B slightly decreased after 4 h of inoculation with serum.

### Efficacy of Peptides in the Mouse Skin Infection Model

**FIGURE 8:**
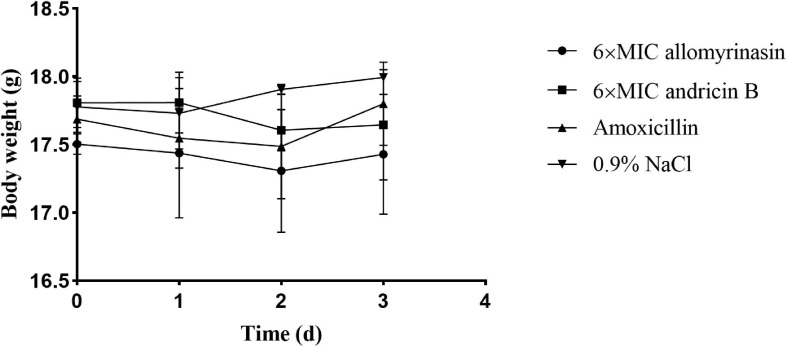
Body weights of mice in different groups treated with allomyrinasin, andricin B, amoxicillin, and saline at 6× minimal inhibitory concentration (MIC) in a mouse skin infection model induced by *Staphylococcus pseudintermedius* MW767056. Data are presented as means ± SD (*n* = 5).

A mouse model of *S. pseudintermedius* skin infection was established. During the observation period, there was no obvious abnormality in food intake, drinking water, or mental state changes of mice treated with low-dose 6× MIC AMPs. There was no significant difference in body weight among the groups (Figure 8).

**FIGURE 9:**
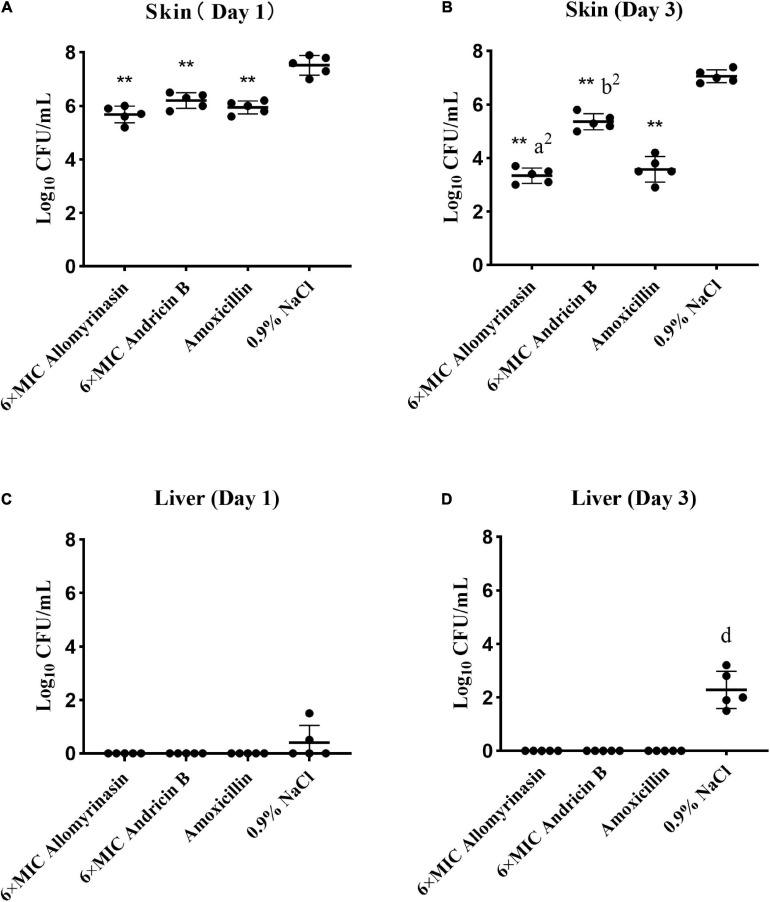
**(A–D)** Efficacy of allomyrinasin and andricin B at 6× minimal inhibitory concentration (MIC) on skin and hepatic infections in a mouse model of *Staphylococcus pseudintermedius* (MW767056) skin infection. The skin was treated daily with allomyrinasin, andricin B, amoxicillin, or 0.9% NaCl after inoculation of 5 × 10^6^ CFU of *S. pseudintermedius* MW767056. The mean bacterial counts (log_10_ CFU/g) were measured on the first and third days after inoculation with *S. pseudintermedius*. Data are presented as means ± SD (*n* = 5). *p < 0.05, **p < 0.01, compared with saline group; ^a^*p* < 0.05, ^a2^*p* < 0.01, compared with group treated with andricin B; ^b^*p* < 0.05, ^b2^*p* < 0.01, compared with group treated with amoxicillin.

Significant differences in skin and hepatic infection prevention were observed between groups treated with antimicrobials and saline. On day 1, all administrations significantly decreased the bacterial load of *S. pseudintermedius* in infected tissues compared with saline (*p* < 0.01, Figure 9A). Specifically, the bacterial counts of *S. pseudintermedius*-infected skin tissue treated with allomyrinasin, andricin B, and amoxicillin were 5.68, 6.2, and 5.94 log_10_ CFU/g, respectively, which were significantly lower than the 7.52 log_10_ CFU/g in the saline-treated group (Figure 9A). No bacterial load was detected in the liver of each AMP and amoxicillin-treated group 1 day after skin infection. However, bacteria were found in the liver homogenate of two mice in the saline-treated group (Figure 9C).

On day 3, further reductions in bacterial counts up to 3.34, 5.36, and 3.58 log_10_ CFU/g in infected skin tissues could be achieved by treatment with allomyrinasin, andricin B, and amoxicillin, respectively, while treatment with saline rose to 7.06 log_10_ CFU/g (*p* < 0.01, Figure 9B). Furthermore, allomyrinasin and amoxicillin were more effective in killing bacteria in infected skin than andricin B, but there was no significant difference in antimicrobial efficacy between allomyrinasin and amoxicillin (allomyrinasin vs. andricin B, *p* < 0.01; andricin B vs. amoxicillin, *p* < 0.01, Figure 9B). Interestingly, treatment with allomyrinasin, andricin B, and amoxicillin completely prevented *S. pseudintermedius* infection in the liver, but in the group treated with saline, liver infections were recorded (Figure 9D). These results indicate that low concentrations of allomyrinasin, andricin B, and amoxicillin can effectively reduce the number of *S. pseudintermedius* in the epidermis of infected mice.

### Pathology and Histological Analysis

**FIGURE 10:**
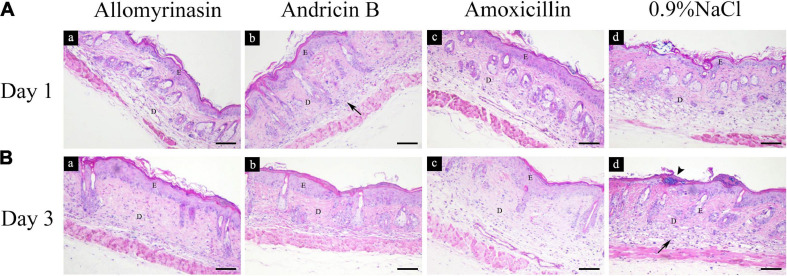
Histopathological analysis of mouse skin tissues infected by *Staphylococcus pseudintermedius* MW767056, evaluated by hematoxylin and eosin staining. **(A)** Inflammatory infiltrate (arrow) was mainly detected in the dermis and scattered in the epidermis on day 1. **(B)** Increased epidermal thickness and an alleviated inflammatory response were observed in all treatment groups, accompanied by staphylococci colonizing the stratum corneum (arrowhead) in the saline-treated group on day 3. The bar represents 100 μm. E, epidermis; D, dermis.

Treatment with AMPs and amoxicillin alleviated inflammation in the infected skin among the treatment groups. An inflammatory infiltrate was mainly observed in the dermis and scattered in the epidermis in all mouse groups on day 1 (Figure 10A). On day 3, the skin of treated mice exhibited increased epidermal thickness but with significantly diminished dermal inflammation, whereas the saline-treated mice displayed a large number of inflammatory cells infiltrating the dermis (Figure 10B).

### Allomyrinasin Inhibited the Proinflammatory Cytokines IL-6 and TNF-α Induced by *Staphylococcus pseudintermedius*

**FIGURE 11:**
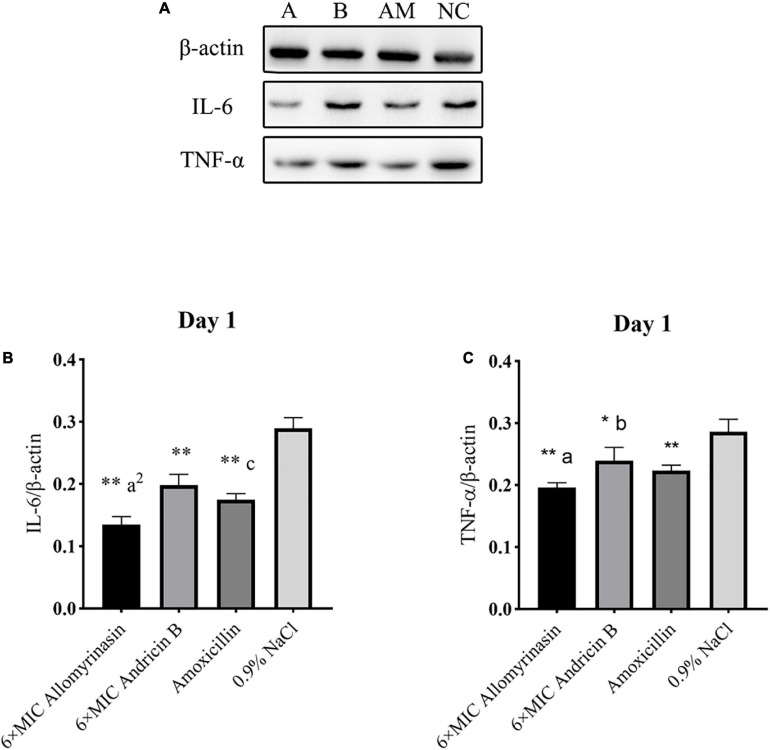
Allomyrinasin inhibited the proinflammatory cytokines IL-6 and TNF-α on day 1 after skin infections induced by *Staphylococcus pseudintermedius* MW767056. **(A)** Western blotting analysis of IL-6 and TNF-α expression in the skin tissues of mice. **(B,C)** Expression of IL-6 and TNF-α, with β-actin as the internal control, in the infected skin is shown as bar graphs. Data with error bars are presented as the mean ± SD (*n* = 3). (A) 6× minimal inhibitory concentration (MIC) allomyrinasin. **(B)** 6× MIC andricin B. AM, amoxicillin; NC, 0.9% NaCl. *p < 0.05, **p < 0.01, compared with saline group; ^a^*p* < 0.05, ^a2^*p* < 0.01, compared with group treated with andricin B; ^b^*p* < 0.05, ^b2^*p* < 0.01, compared with group treated with amoxicillin.

**FIGURE 12:**
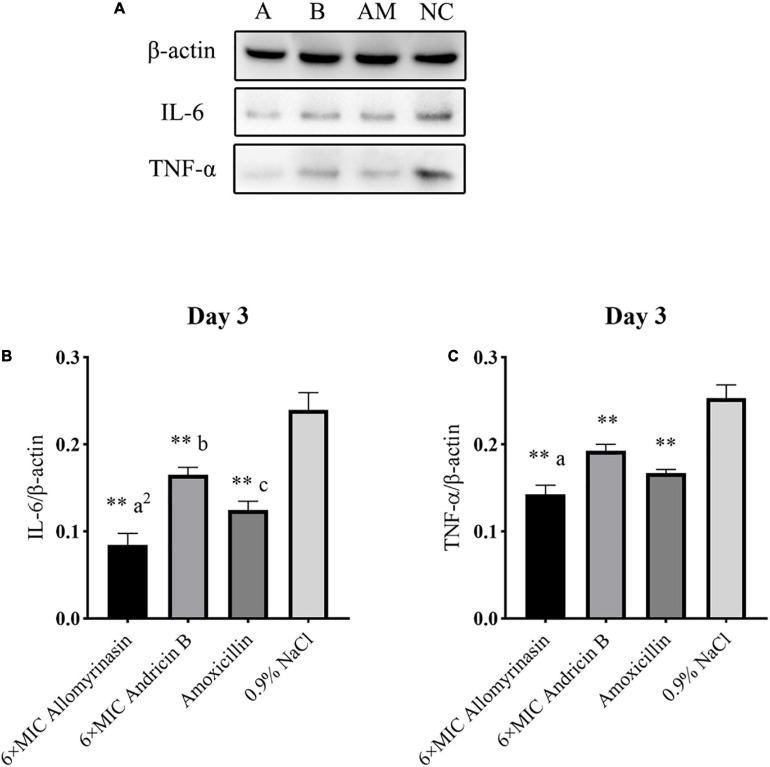
Allomyrinasin inhibited the proinflammatory cytokines IL-6 and TNF-α on day 3 after skin infections induced by *Staphylococcus pseudintermedius* MW767056. **(A)** Western blotting analysis of IL-6 and TNF-α expression in the skin tissues of mice. **(B,C)** Expression of IL-6 and TNF-α, with β-actin as the internal control, in the infected skin is presented in bar graphs. Data with error bars are presented as the mean ± SD (*n* = 3). **(A)** 6× minimal inhibitory concentration (MIC) allomyrinasin. **(B)** 6× MIC andricin B. AM, amoxicillin; NC, 0.9% NaCl. *p < 0.05, **p < 0.01, compared with saline group; ^a^*p* < 0.05, ^a2^*p* < 0.01, compared with group treated with andricin B; ^b^*p* < 0.05, ^b2^*p* < 0.01, compared with group treated with amoxicillin.

The antimicrobial and anti-inflammatory properties of the tested peptides were investigated via quantification of the expression levels of the proinflammatory cytokines TNF-α and IL-6 by Western blotting (Figures 11A, 12A).

On day 1, the expression level of TNF-α protein in each treated group decreased, and there was a significant difference between the treated groups and the saline-treated group (allomyrinasin vs. 0.9% NaCl, *p* < 0.01; andricin B vs. 0.9% NaCl, *p* < 0.05; amoxicillin vs. 0.9% NaCl, *p* < 0.01, Figure 11C). These results indicate that the tested peptides at low concentrations can effectively reduce the expression of TNF-α in infected mice. The expression of TNF-α protein in the group treated with allomyrinasin and amoxicillin was significantly lower than that in the group treated with andricin B (allomyrinasin vs. andricin B, *p* < 0.05; amoxicillin vs. andricin B, *p* < 0.05, Figure 11C). Similarly, the protein expression level of IL-6 in each treated group decreased, and there was a significant difference between the treated groups and the saline-treated group (*p* < 0.01, Figure 11B). Furthermore, the protein expression of IL-6 in the allomyrinasin-treated group was significantly lower than that in the groups treated with andricin B and amoxicillin (allomyrinasin vs. andricin B, *p* < 0.01; allomyrinasin vs. amoxicillin, *p* < 0.05, Figure 11B).

On day 3, the protein expression of TNF-α in each group was further reduced, with a significant difference between the treated groups and the saline-treated group. Additionally, the expression of TNF-α protein in the allomyrinasin group was significantly lower than that in the andricin B group (*p* < 0.05, Figure 12C). Similarly, the protein expression level of IL-6 in the group treated with peptides and antibiotics significantly decreased compared with that in the saline-treated group (*p* < 0.01, Figure 12B). Meanwhile, the expression of TNF-α in the group treated with allomyrinasin was significantly lower than that in the groups treated with andricin B and amoxicillin (allomyrinasin vs. andricin B, *p* < 0.01; allomyrinasin vs. amoxicillin, *p* < 0.05), and the expression of IL-6 protein in the group treated with andricin B was significantly higher than that in the group treated with amoxicillin (*p* < 0.05, Figure 12B).

## Discussion

In the present study, the antimicrobial activities of the selected short synthetic peptides and their potential for animal application were evaluated.

Allomyrinasin is a cationic peptide that exhibits a broad spectrum of antibacterial and antifungal activities (Lee et al., 2019). In the present study, we observed that allomyrinasin displayed potent antibacterial activities against the clinical isolates of *S. pseudintermedius* and *S. cohnii*, while andricin B showed moderate activity. It should be emphasized that all tested peptides demonstrated powerful antifungal activities against the zoonotic fungi, *M. canis*, *T. mentagrophytes*, and *M. gypseum*, the most common dermatophytes with the high potential for zoonotic infections by contact with animals (Cafarchia et al., 2006).

It is universally known that the structure–function relationship of AMPs is largely determined by numerous physicochemical properties of peptides, including charge, hydrophobicity, sequence, and size (Yeaman and Yount, 2003). Among them, the biological activity and specificity of AMPs are closely associated with cationicity. Cationic AMPs are initially bound to the bacterial cytomembrane by the mean of electrostatic force between cationic amino acid residues within AMPs and negatively charged phospholipids of the bacterial cytomembrane. The cationic charge of AMPs strongly correlates with their activity as the capability of binding to the membrane interface began to improve with an increase in net positive charge (Bonucci et al., 2014; Fillion et al., 2015). Allomyrinasin and andricin B are both cationic AMPs with net positive charge of +3 and +2, respectively. The stronger effect of allomyrinasin on bacterial strains and erythrocytes than andricin B may be attributed to its higher net positive charge. Theoretically, AMPs with more cationic residues should exhibit more robust antimicrobial activity; however, there is an optimum charge beyond which higher or lower cationicity can considerably decrease their biological activity. The addition of cationic residues of magainin 2 resulted in increased hemolysis and the decreased selectivity for microbial cells (Dathe et al., 2001). Similarly, removal of the cationic C-terminal residues of melittin also led to a significant drop in hemolytic activity and membrane binding ability (Hall et al., 2011; Wang et al., 2015). In addition, hydrophobicity has been considered as another crucial parameter to regulate the antimicrobial potency and hemolytic capacity of AMPs. Hydrophobicity plays a significant role in interaction with cell membranes by impacting the extent to which AMPs are capable of penetrating into the membrane layer. Similar to charge, modulating hydrophobicity to an optimal percentage can boost antimicrobial activity against microbial cytomembranes, but once beyond this optimum point, impaired antimicrobial activity and increased mammalian cytotoxicity can appear (Pasupuleti et al., 2012). In our study, although allomyrinasin possessed the same amount of hydrophobic residues with andricin B, it may possess an optimum balance between cationicity and hydrophobicity, as indicated by the more potent antimicrobial efficacy and biofilm inhibition activity with acceptable mammalian hemolysis (Wimley, 2010).

Rapid antibiosis at the epidermis is pivotal for the recovery of skin barrier function and is capable of inhibiting the emergence of drug resistance and preventing infection spreading (Finberg et al., 2004). *In vitro* time-kill kinetics revealed complete and rapid eradication of *S. pseudintermedius*, *S. cohnii*, and *M. canis* within hours in a time- and concentration-dependent manner. This rapid antimicrobial elimination by AMPs may display great suitability for animal applications and may result in better treatment outcomes than conventional antimicrobials.

Allomyrinasin and andricin B exhibited antibacterial patterns similar to membrane-lytic peptides, such as the antibiotic nisin, rather than nonlytic peptides (Park et al., 1998), identified by bacteriolysis analysis of *S. pseudintermedius* exposed to peptides. These figures indicated that disrupting bacterial membrane integrity is the underlying mechanism of action. Furthermore, we investigated the action of peptides on *S. pseudintermedius* membranes using calcein leakage assays for validation. Interestingly, both allomyrinasin and andricin B exhibited cytomembrane permeabilization in a dose- and time-dependent manner, which is similar to the data arising from the well-studied membrane-dissolving peptide nisin. These findings clearly illustrated that the accomplishment of bacterial lysis by peptides is attributable to pore formation, membrane permeabilization, and cytoplasmic leakage.

Microbial biofilms, constituting a thorny issue facing current antimicrobial agents, are pivotal in the pathogenic mechanism of skin and wound infections. Clinical treatment failure usually results from impediment by biofilms to penetration of antimicrobials to microbes (Batoni et al., 2021). Actively metabolizing cells are the main target of traditional therapeutics rather than quiescent cells due to inhibition of essential biomacromolecule synthesis in these growing cells (Hurdle et al., 2011). However, the capability of some AMPs to directly disrupt the cellular membranes of microbes, a cellular structure in both proliferative and quiescent cells, is anticipated to be more efficacious in biofilm eradication (Nuti et al., 2017). In our study, allomyrinasin was shown to inhibit *S. pseudintermedius* and *S. cohnii* biofilm formation and disrupt mature biofilms efficiently, while andricin B and pinipesin exhibited moderate activity with relatively high values of MBIC and MBEC. Furthermore, biofilm formation also plays a significant role in the pathogenesis of dermatophytosis (Sherry et al., 2017). Notably, allomyrinasin was highly efficient in impeding *M. canis* biofilm formation and in clearing mature biofilms, while andricin B and pinipesin were relatively ineffective.

Since the ability of peptides to hinder microbial growth alone was evaluated, we then investigated their synergistic ability with other traditional antimicrobials. Amoxicillin is a widely applied antibiotic that is capable of blocking the synthesis of glycopeptides, a significant component of staphylococcal cell walls, by specifically inactivating transpeptidase in bacteria (de Marco et al., 2017). Terbinafine is a allylamine antifungal agent frequently used in the treatment of the superficial fungal infections. It exclusively inhibits squalene oxygenase in the process of ergosterol biosynthesis, leading to excessive accumulation of squalene in fungal cells and cell death. It has been reported that amoxicillin and terbinafine are widely applied in the treatment of bacterial and fungal infections; however, these pathogens have evolved resistance by different mechanisms (Ghannoum, 2016; Monod and Méhul, 2019; de Jong et al., 2020; Wegener et al., 2020). In the present study, we observed a synergistic relationship between tested peptides and conventional antimicrobials, as indicated by complete clearance of *S. pseudintermedius*, *S. cohnii*, and *M. canis* within extremely low concentrations. We speculated that the synergistic effect between peptides and antibiotics may be ascribed to the inhibition of the bacterial cell wall synthesis by amoxicillin, allowing more peptides to access the bacterial membrane.

One of the primary limitations of the application in the treatment of skin and wound infections is inactivation of AMPs by serum either through protease cleavage or protein binding (Yeung et al., 2011). In our study, allomyrinasin and andricin B are capable of maintaining their antimicrobial activity at different ratios in the presence of 25% FBS. Their capacity to withstand degradation in serum provides a potential for administration of peptides in physiological solutions. Hemolysis is another noticeable obstacle in antimicrobial development, especially when the cell membrane acts as the drug target. The discovery of clinically feasible AMPs has been hindered by unexpected cytotoxicity to eukaryotic cells, particularly erythrocytes at therapeutic dosages (Edwards et al., 2017). The selectivity of AMPs can be indicated by the therapeutic index, defined as the ratio between MHC and MIC (MHC/MIC). AMPs with the higher therapeutic index often exhibit more effective activity as an antibiotic (Maturana et al., 2017). In our study, allomyrinasin with a therapeutic index in the range from 16 to 32 can be considered as a more effective antimicrobial than andricin B with a therapeutic index of 16.

The results from *in vitro* antimicrobial assays laid a solid groundwork for further research on animals. In this study, the anti-infective and anti-inflammatory effects of the peptides were assessed using a mouse skin infection model. Microbial clearance is the ultimate objective of anti-infectious treatments, with bacterial burden being the major determinant of therapeutic effects (Ball et al., 2002). Daily topical treatment with allomyrinasin and andricin B at a low concentration (6× MIC) efficiently decreased the skin bacterial load in a time-dependent fashion and achieved complete prevention of hepatic infection, whereas liver colonization appeared in 20% and 100% of mice after 1 and 3 days of treatment with saline, respectively. Compared with amoxicillin, andricin B was less effective (*p* < 0.01) in clearing the bacterial load in infected skin, while allomyrinasin showed a similar efficacy (*p* > 0.05). Based on the outstanding synergism between allomyrinasin and amoxicillin exhibited by *in vitro* assays, the combination therapy may provide an ideal option for treatment of skin infections in the context of animal husbandry and medicine. However, complete removal of *S. pseudintermedius* from skin was not realized by the applied antimicrobials, indicating a requirement for prolonging treatment duration.

The skin infections induced by *S. pseudintermedius* can be aggravated by excess expression of host proinflammatory factors more than by bacterial load. An exacerbated inflammatory response not only largely defers wound healing but also is likely to induce cicatrization. AMPs with integrated microbicidal and immunomodulatory abilities are capable of promoting epithelialization and wound healing and should occupy a dominant position in the treatment of *S. pseudintermedius* skin infections (Mangoni et al., 2016). Inflammatory reactions in infected skin were observed in the entire pathological process as anticipated due to manual scratch and *S. pseudintermedius* infection. However, with the increase of the medication duration, the protein expression of inflammatory factors (TNF-α and IL-6) in the antimicrobial-treated groups significantly decreased compared with those in the saline-treated group (*p* < 0.01), indicating the alleviation of the inflammatory response. In particular, allomyrinasin seemed to be superior in anti-inflammatory efficacy when compared with two other antibacterial agents, which can be attributed to its potent antibacterial ability *in vitro* and high stability in physiological solutions, such as serum (Figure 6).

Although AMPs have exhibited the unique anti-infective actions and there is an emergent need for new antimicrobials, they have previously been disregarded due to high production costs (Mulani et al., 2019). However, the remarkable reductions in the excessive manufacturing cost of AMPs have been achieved due to technological progress and wide application of solid-phase peptide synthesis. These newly synthesized peptides exhibited microbicidal potency with a unique mechanistic action, robust stability, biofilm formation prevention, and mature biofilm-eradicating effectiveness, as well as synergism with conventional antimicrobials. Topical application significantly reduced the wound bacterial load, prevented hepatic dissemination, and alleviated the skin inflammatory response. In conclusion, the clinical application of allomyrinasin may provide an ideal option for treating animal skin infections, particularly in the context of medicine and animal husbandry, despite additional investigation required to optimize pharmaceutical formulation and delivery.

## Data Availability Statement

The datasets presented in this study can be found in online repositories. The names of the repository/repositories and accession number(s) can be found below: https://www. ncbi.nlm.nih.gov/nuccore/?term=MW767025:MW767027[accn], MW767025-MW767027; https://www.ncbi.nlm.nih.gov/nuccore/MW766984, MW766984; https://www.ncbi.nlm.nih.gov/nuccore/MW768151, MW768151; https://www.ncbi.nlm.nih.gov/nuccore/MW766983, MW766983; https://www.ncbi.nlm.nih.gov/nuccore/?term=MW793383:MW793392[accn], MW793383-MW793392; https://www.ncbi.nlm.nih.gov/nuccore/MW767046, MW767046; and https://www.ncbi.nlm.nih.gov/nuccore/?term=MW767051: MW767056[accn], MW767051-MW767056.

## Ethics Statement

The animal study was reviewed and approved by Animal Care and Use Committee of China Agricultural University.

## Author Contributions

QT and DL contributed conception and design of the study. QT, YZ, and XW organized the database, performed the statistical analysis, and wrote the first draft of the manuscript. QT, CY, and WL wrote sections of the manuscript. All authors contributed to manuscript revision, read, and approved the submitted version.

## Conflict of Interest

ZM was employed by the company Artron BioResearch Inc. The remaining authors declare that the research was conducted in the absence of any commercial or financial relationships that could be construed as a potential conflict of interest.

## Publisher’s Note

All claims expressed in this article are solely those of the authors and do not necessarily represent those of their affiliated organizations, or those of the publisher, the editors and the reviewers. Any product that may be evaluated in this article, or claim that may be made by its manufacturer, is not guaranteed or endorsed by the publisher.
