# Supplementary material for: Evaluation of Short-Chain Antimicrobial Peptides With Combined Antimicrobial and Anti-inflammatory Bioactivities for the Treatment of Zoonotic Skin Pathogens From Canines
Source: Front Microbiol. 2021 Aug 11;12:684650. doi: 10.3389/fmicb.2021.684650 (PMC8386128; doi:10.3389/fmicb.2021.684650)
Supplement: Supplementary file 1 [file Table_1.DOCX]

**TABLE 1**. **Accession numbers for bacterial 16S rRNA genes and fungal nuclear rDNA-ITS genes.**

| Strains | GenBank accession number |
| --- | --- |
| *S. pseudointermedius* SP1 | MW767056 |
| *S. pseudointermedius* SP2 | MW793383 |
| *S. pseudointermedius* SP3 | MW793384 |
| *S. pseudointermedius* SP4 | MW793385 |
| *S. pseudointermedius* SP5 | MW793386 |
| *S. pseudointermedius* SP6 | MW793387 |
| *S. pseudointermedius* SP7 | MW793388 |
| *S. pseudointermedius* SP8 | MW793389 |
| *S. pseudointermedius* SP9 | MW793390 |
| *S. pseudointermedius* SP10 | MW793391 |
| *S. pseudointermedius* SP11 | MW793392 |
| *S. pseudointermedius* SP12 | MW767046 |
| *S. cohnii* | MW767053 |
| *S. haemolyticus* | MW767051 |
| *S. sciuri* | MW767055 |
| *S. simulans* | MW767052 |
| *P. mirabilis* | MW767054 |
| *M. canis* QX1 | MW767025 |
| *M. canis* QX2 | MW767026 |
| *M. canis* QX3 | MW767027 |
| *M. canis* QX4 | MW768151 |
| *T. mentagrophyte* | MW766984 |
| *M. gypseum* | MW766983 |
